# Cardiac involvement of diffuse large B‐cell lymphoma presenting as various arrythmias

**DOI:** 10.1002/ccr3.6504

**Published:** 2022-11-19

**Authors:** Tadakiyo Ido, Hitoshi Minamiguchi, Yasuharu Ichibori, Takaharu Hayashi, Nobuhiko Makino, Atsushi Hirayama, Yoshiharu Higuchi

**Affiliations:** ^1^ Department of Cardiology Osaka Police Hospital Osaka Japan

**Keywords:** autopsy, cardiac involvement, diffuse large B‐cell lymphoma, malignant lymphoma, sick sinus syndrome

## Abstract

Symptomatic cardiac involvement of malignant lymphoma is rare. Silent invasion of malignant lymphoma makes it difficult to diagnose it in the early phase of clinical course. We describe a case with cardiac involvement of diffuse large B‐cell lymphoma presenting various types of arrythmias that were not diagnosed until autopsy.

## Graphical abstract

Symptomatic cardiac involvement of malignant lymphoma is quite rare. Furthermore, silent and rapid invasion of malignant lymphoma makes it difficult to diagnose it in the early phase of clinical course. Once detecting various types of arrythmias, we should have suspected of these kinds of infiltrated tumors.

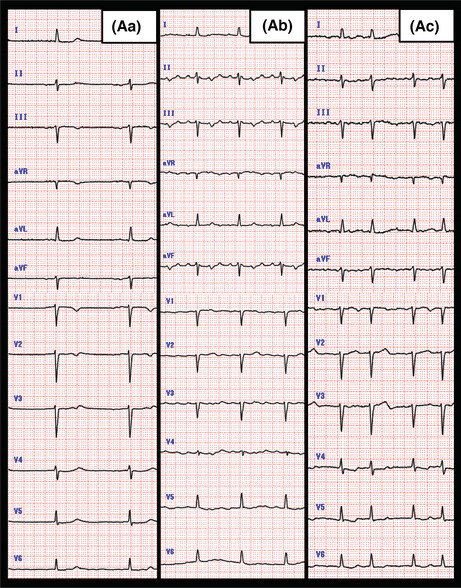

## 1 CASE IMAGES

**FIGURE 1:**
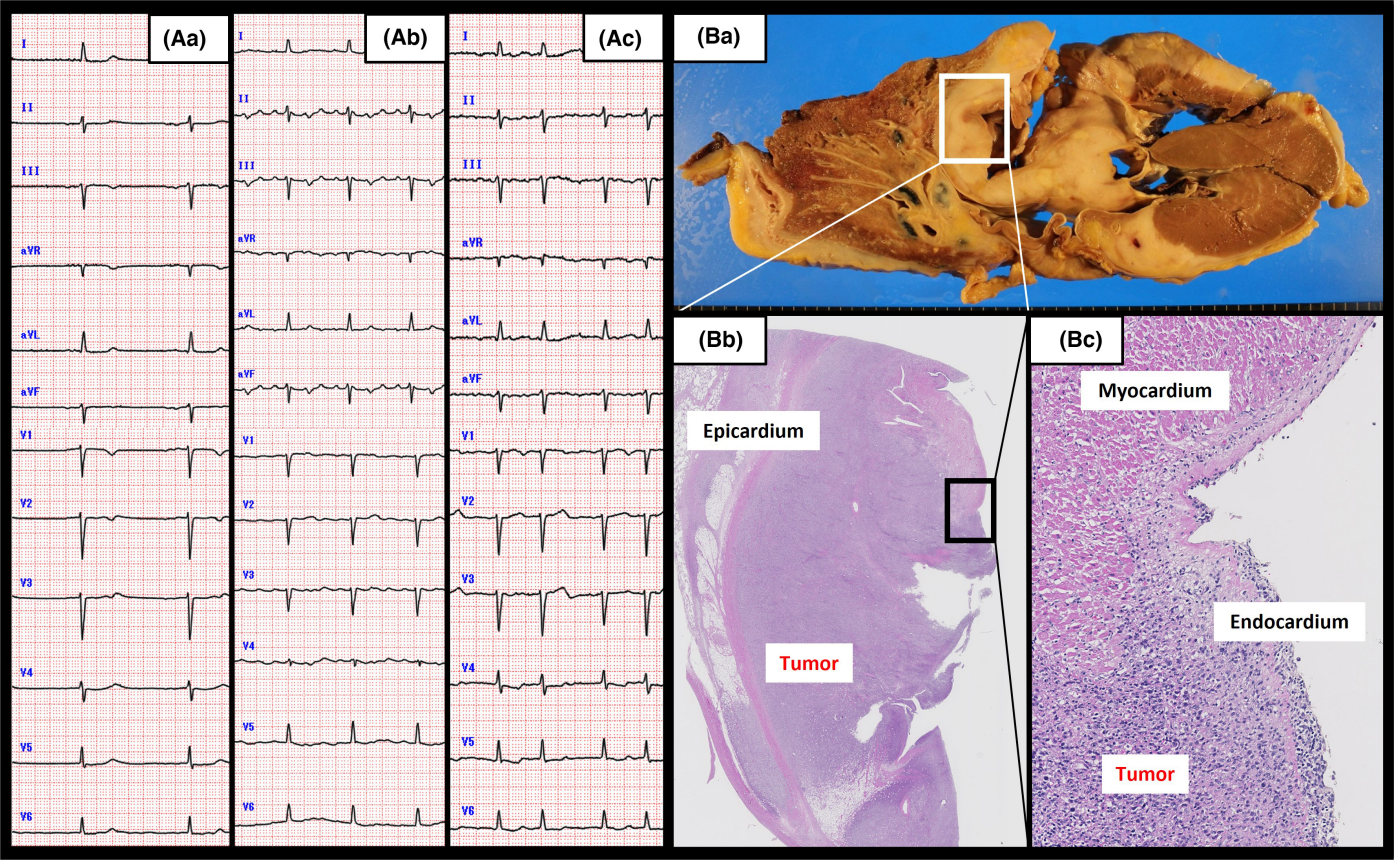
(A–a) Ectopic atrial rhythm. (A–b) Atrial tachycardia with 3:1 conduction. (A–c) Atrial fibrillation. (B–a) The widespread malignant tumor appeared as a light‐yellow color. (B–b, c) Histology showing that the malignant tumor cells had invaded a large amount of the right atrium (hematoxylin–eosin stain, magnification ×40, ×100).

An 84‐year‐old male patient with a history of coronary artery disease presented to our institution with an unknown fever and vomiting. No specific findings were observed in echocardiography and CT image, however, his electrocardiogram demonstrated 43 bpm of ectopic atrial rhythm without sinus rhythm (Panel A‐a). We thought the sinus arrest was caused by hyperkalemia (potassium of 6.9 mEq/L) followed by worsening renal function (blood urea nitrogen 84.6 mg/dl and creatinine 4.77 mg/dl). We immediately placed a temporary pacemaker and performed continuous hemodiafiltration; however, regardless of multidisciplinary treatment, he died on the thirty‐eighth day of admission. His autopsy revealed cardiac involvement of diffuse large B‐cell lymphoma (DLBCL). The diffuse infiltrated large lymphoid cells were observed to have right atrial dominance (Panel B). In this clinical course, the electrocardiogram demonstrated atrial tachycardia with 3:1 conduction and atrial fibrillation (Panel A–b, c). Both tachycardias were followed by sinus arrest. The patient presented with various types of arrhythmias such as sick sinus syndrome, atrial tachycardia, and atrial fibrillation. Theoretically, the infiltrated DLBCL invaded the sinus node and right atrium, then induced these various arrhythmias in this case. Alternatively, cardiac involvement of malignant lymphoma is usually non‐symptomatic.^1^ It is important not to dismiss a rare symptomatic case of cardiac involvement of malignant lymphoma since there were various types of arrhythmias that might have been treated with chemotherapy.^2^ Once detecting various types of arrythmias, we should have suspected of these kinds of infiltrated tumors (Figure 1).

## AUTHOR CONTRIBUTIONS

TI and HM contributed to treat the patient and drafted the manuscript. YI, TH, and NM contributed to diagnose and treat the patient. AH and YH critically reviewed the literature and involved in writing. All authors approved the final manuscript.

## CONFLICT OF INTEREST

None declared.

## CONSENT

Written informed consent was obtained from the patient to publish this report in accordance with the journal's patient consent policy.

## Data Availability

The data that support the findings of this study are available from the corresponding author upon reasonable request.
